# Potential Values of Circulating microRNA-21 to Predict Early Recurrence in Patients with Colorectal Cancer after Treatments

**DOI:** 10.3390/jcm11092400

**Published:** 2022-04-25

**Authors:** Yun-Jie Hao, Chih-Yung Yang, Ming-Hsien Chen, Lu-Wey Chang, Chien-Ping Lin, Liang-Chuan Lo, Sheng-Chieh Huang, You-You Lyu, Jeng-Kai Jiang, Fan-Gang Tseng

**Affiliations:** 1 Department of Engineering and System Science, National Tsing Hua University, Hsinchu 30013, Taiwan; hyjtb2009@gmail.com (Y.-J.H.); mhchentwww@gmail.com (M.-H.C.); xxo831230@gmail.com (L.-W.C.); 2 School of Engineering, University of Liverpool, Liverpool L69 3BX, UK; 3 Department of Teaching and Research, Taipei City Hospital, Taipei 10341, Taiwan; yc3636@hotmail.com; 4 Commission for General Education, National United University, Miaoli 36003, Taiwan; 5 General Education Center, University of Taipei, Taipei 110014, Taiwan; 6 Institute of Microbiology and Immunology, National Yang-Ming Chiao-Tung University, Taipei 11221, Taiwan; jainping@gmail.com (C.-P.L.); noni19910124@gmail.com (L.-C.L.); yylyu14@gmail.com (Y.-Y.L.); 7 School of Medicine, National Yang-Ming Chiao-Tung University, Taipei 11221, Taiwan; schuang5@vghtpe.gov.tw; 8 Department of Surgery, Division of Colorectal Surgery, Taipei Veterans General Hospital, Taipei 11217, Taiwan; 9 Department of Engineering and System Science, Frontier Research Center on Fundamental and Applied Sciences of Matters, National Tsing-Hua University, Hsinchu 30013, Taiwan; 10 Research Center for Applied Sciences, Academia Sinica, No. 128, Sec. 2, Academia Rd., Nankang, Taipei 11529, Taiwan

**Keywords:** plasma microRNA21 (miR-21), exosomal microRNA21 (exo-miR-21), circulating tumour cells (CTCs), circulating tumour microemboli (CTM), carcinoembryonic antigen (CEA), carbohydrate antigen 19-9 (CA19-9), colorectal cancer (CRC), peripheral blood (PB), prognosis, recurrence

## Abstract

Insufficient prognosis of local recurrence contributes to the poor progression-free survival rate and death in colorectal cancer (CRC) patients. Various biomarkers have been explored in predicting CRC recurrence. This study investigated the expressions of plasma/exosomal microRNA-21 (miR-21) in 113 CRC patients by qPCR, their values of predicting CRC recurrence, and the possibility to improve the prognostic efficacy in early CRC recurrence in stratified patients by combined biomarkers including circulating miR-21s, circulating tumour cells/microemboli (CTCs/CTM), and serum carcinoembryonic antigen (CEA)/carbohydrate antigen 19-9 (CA19-9). Expressions of plasma and exosomal miR-21s were significantly correlated (*p* < 0.0001) in all and late-stage patients, presenting similar correlations with other biomarkers. However, stage IV patients stratified by a high level of exosomal miR-21 and stage I to III patients stratified by a high level of plasma miR-21 displayed significantly worse survival outcomes in predicting CRC recurrence, suggesting their different values to predict CRC recurrence in stratified patients. Comparable and even better performances in predicting CRC recurrence in late-stage patients were found by CTCs/CTM from our blood samples as sensitive biomarkers. Improved prognosing efficacy in CRC recurrence and better outcomes to significantly differentiate recurrence in stratified patients could be obtained by analysing combined biomarkers.

## 1. Introduction

Colorectal cancer (CRC) is one of the most common cancers diagnosed in humans worldwide, and for years, it has been among the top three leading causes of death in cancer patients [1]. Recommended clinical screening tests such as the colonoscopy (gold standard), fecal immunochemical test, fecal occult blood test, and sigmoidoscopy are helpful in reducing mortality generally, but disadvantages such as invasive properties, expensive prices, and poor sensitivity and specificity constrain the range of their applications and lower the rates of participation, compliance, and adherence from patients and people who are eligible and are suggested to check regularly [2]. Owing to the development of new screening strategies, such as computed tomographic (CT) colonography and colon capsule endoscopy (CCE) [3,4], advancements in therapeutic technologies such as chemotherapy and radiotherapy and discoveries of various molecular signatures, including gene or protein mutants, integrities, DNA methylation, fecal microbiota, and novel biomarkers in various bodily fluids, have gradually improved both diagnosis and curative treatment for CRC patients in recent decades. However, prognosis and survival rate remain low, with poor amelioration in the follow-ups after patients’ first clinical interventions [5,6]. In addition to reasons such as slow progress in tumorigenesis, inflammatory responses of the immune system to cancer and treatment, and dysfunctions of other organs such as the liver, drawbacks and challenges of currently employed biomarkers partially contribute to the often failed and ineffective detection of early incident, local recurrence, and distant metastasis in CRC patients, comprehensively leading to insufficient prognosis and poor survival rate but high metastasis and metastatic mortality in CRC patients, especially for those who had stage IV (advanced, high risk) CRC within five-year follow-up after treatments [2,7,8].

Therefore, non-, or minimal invasive, more cost-effective, highly stable, robust, and accurate screening modalities and biomarkers for CRC are needed urgently [8] and are important for clinicians to perform early diagnosis, continuous tracking, and prospective prognosis at an early time of the follow-up after first treatment. Pre- and post-operative surveillances such as responsive benefit or resistance from patients to past or future therapies as well as potential recurrence or metastatic incidents would also be predicted more accurately to improve risk management, proper patient stratification, and survival rates in CRC cases on further treatment [6].

Current biomarkers in liquid biopsies, including circulating tumour cells (CTCs) and microemboli (CTM), plasma/serum microRNAs, extracellular vesicle (EVs), especially the exosomes derived from cancer cells or CTCs, and the exosomal microRNAs (exo-miRNAs), have recently been explored as important participants in the pathological process of various cancers [9,10,11,12,13], including CRC [14,15,16,17,18,19,20,21,22], due to direct or indirect associations between their biogenesis and primary or secondary tumours [9,23,24]. 

As rare malignant tumour cells shed from primary or secondary (metastatic sites) solid tumours [25,26] into the blood circulation system, CTCs are seen as valuable biomarkers providing information about disease progress, patient recurrence, and survival situations, especially in advanced and metastatic cases and potential responses to treatments such as chemotherapy and outcomes from patients [14,27,28,29,30]. CTM was observed in CTC studies and was also previously called circulating micrometastases or CTC clusters [31], which were often depicted as the metastatic population of heterogeneous multicellular aggregates containing about 3 to 100 cells. Comparing to single CTC, CTM possesses highlighted features of stemness and immune evasion, higher resistance to shear stress, and anoikis, which is more relevant to both metastasis and anti-apoptosis. The size, morphology, frequency, and cellular and molecular profiles of CTM are not fully understood but are worthy of clinical studies, especially its correlations with overall survival (OS), progression-free survival (PFS), metastasis and the prognostic value in various types of cancers [12,32]. Although it is not quite accessible for people to accurately detect CTM in liquid biopsies due to such issues, as the vulnerability of cell clusters, limited devices and technologies to visualise, identify and capture CTM in vitro, as well as availability of clinical signatures for study [12], increasing knowledge of CTCs/CTM has accumulated valuable information such as their biogenesis and primary tumour molecular hallmarks, metastatic distribution and various signalling pathways involving the proliferation, adhesion, stemness, immune activation, apoptosis, and migration (epithelial to mesenchymal transition and reversely) of cancer cells [12]. 

MicroRNAs (miRNAs), as a class of small non-coding single-stranded RNA molecules with a length ranging from 18 to 25 nucleotides, act on regulating messenger RNAs (mRNAs) post-transcriptionally by using complementary sequences [33]. Since first discovered in C. elegans decades ago [34,35], a huge number of miRNAs and their gene targets in plant and animal, tissues, and cells, have been identified. Their dysfunctions or changes in sequences or expression level are involved in numerous pathogeneses of human diseases, in particular cancers [36], where the miRNAs participate in regulating cancer-related genes of many critical processes, from initiation, progression, and recurrence to metastasis, as oncogenes or suppressors of tumours [37], depending on their targets. Given these important functions, many studies have investigated the biosynthesis, action mechanisms, expression profiles and clinical applications of miRNAs as biomarkers for diagnosis, prognosis, assessing medical outcome, and being considered as potential treatment in cancers [38,39,40]. Among various miRNAs, miRNA-21 and the putative carrier of miRNAs, namely the cancer cell-released exosomes identified in liquid biopsies such as serum and plasma of the blood circulation system, were stressed in CRC studies [13,23,41,42]. The highly conservative human miR-21 gene is located on the 17q23-1 chromosome. From experiments in cell lines and dissected tissue samples, the effects of miR-21 on the proliferation and apoptosis of various types of cells including colon cancer cells, were observed, such as direct relationships between the expression of miR-21, and the metastasis and invasion of tumours in CRC patients. Several studies further reported the differences and changes in the expression level of miRNAs, including miR-21 in plasma between CRC patients and healthy people, as well as those in patients before and after treatment, suggesting a significant correlation between CRC and the upregulation of miR-21 expression in diseased tissue and liquid biopsy, in other words, the diagnostic role of miR-21 [43]. The prognostic role of miRNAs such as miR-21 was also displayed in several other studies, indicating significant associations between miR-21 expression and some clinicopathological parameters such as the sites and depth of invasions detected, status of recurrence, progressive disease and metastasis [8,44], TNM [18] and Duke’ stages of cancer [15], especially the high risk or stage IV cases [8,45].

Conversely, as the newly confirmed intercellular communicator in a multicellular system, nano-scaled (30–100 nm) exosomes selectively enclosing various bio-active molecules, such as proteins, lipids, DNA, small interference RNAs (siRNAs), mRNAs, and microRNAs in their lipid bilayer membranes, are functionally originated from the endosomal network of living cells [46] and are usually matured and sorted by the mechanism called the endosomal sorting complexes required for transport (ESCRT) to different destinations [47] before being released or secreted. Their contents reflect the molecular architecture of parent cells [48] and would influence the recipient cells epigenetically [47]. It was also found that almost every type of cell could release exosomes via a highly controlled process, including cancer cells [46]. These cancer cell-derived exosomes have been observed in various types of bodily fluids from cancer patients [48,49]. Increasing evidence has demonstrated them as oncogenic carriers of bio-active cargoes and information transporters inside the tumour microenvironment (TME) or between TME and the surrounding/distant normal sites inside cancer patients [1]. The cancer-related exosomes contribute to modulating many critical processes in cancers such as angiogenesis, extracellular matrix remoulding, and immune responses [48], by which the initiative, development, and progression of the neoplastic diseases could be driven, the normal stromal cells could be modulated, and the pre-metastatic niche could be established and promoted [46]. Based on analyses such as proteomics, biochemical tests, microarray, and next-generation sequences (NGS) [50,51,52], some organ-specific and function-related markers from the cell-dependent exosomes derived from specific types of diseases, especially various cancers, have been characterised [48], further boosting the potential of using exosomes as biomarkers for cancers, although challenges of the isolation, purification, origination, and identification of the cancer-specific or TME-derived exosomes remain and more knowledge about the biological functions of them are required [42,47,48]. In addition to the organ-dependent surface markers as antigens and cancer-specific protein signatures, the exo-miRNAs selectively packaged in the exosomes are more stable and retrospectively detectable than those identified directly from liquid biopsies, providing more attractive and prudent information in cancer studies [48]. 

When considering conventional tumour biomarkers employed in liquid biopsies to regularly screen cancers in clinic, such as the CA15-3 (breast cancer), CA19-9 (pancreatic cancer and CRC), CA125 (ovarian cancer), CEA (CRC) and prostate-specific antigen (prostate cancer), certain conditions are required, including abundant protein expression levels, high-affinitive detection reagents, and high or late stages of cancer samples, whereas low specificity, weak sensitivity, and poor accurate rates were often displayed [10]. In contrast, promising circulating biomarkers such as CTCs, CTMs, miRNAs and exosomes could offer complementary advantages such as ready accessibility, potential of personalised biomarker, abundancy, stability, easy measurement in fluids of the organism and cost-effectiveness. Results from a few recent studies have suggested that to analyse a panel of biomarkers would provide better outcomes [14,27,53,54,55,56,57]. Hence, in this paper, we first investigated the expressions of the circulating miR-21 from both plasma and corresponding plasma-derived exosome, being isolated from a total of 113 peripheral blood (PB) samples of CRC patients, by qPCR. Meanwhile, both numbers of the EpCAM-positive CTC and the presence of CTM from blood samples were detected and analysed via our rare-cell detecting platform and patent self-assembled cell array chip (SACA) [14,27]. By stratifying patients, the distribution, correlation, survivals, recurrence rate and odds ratio of several valuable biomarkers in CRC, including the circulating miRNA-21s, the numbers of CTC, the presence of CTM, the level of serum CEA and CA 19-9, were assessed for the potential to improve the prognosis of recurrence in CRC patients at an early time of follow-up after first clinical treatment.

## 2. Materials and Methods

### 2.1. Enrolment of Clinical Patients and Healthy Volunteers

The protocols of enrolling clinical patients and healthy volunteers in this study were performed and registered under the framework of the Institutional Review Board (IRB-number: 2017-07-008CC) of Taipei Veterans General Hospital and NTHU (IRB-numbers: 11010HE119). All patients and healthy volunteers included in this study agreed to and signed their informed consents.

### 2.2. Plasma Preparation, CTC/CTM Detection, Exosome Isolation and Total microRNAs Extraction from Plasma and Exosome

#### 2.2.1. Plasma Preparation and CTC/CTM Detection

Peripheral blood (PB) samples were collected before treatment in a collaborative hospital (Taipei Veterans General Hospital) and were delivered to our lab in NTHU within 24 h to perform plasma preparation and CTC/CTM enumeration.

Plasma of every sample was prepared during the pre-processing steps as mentioned in previous studies [14,27]. In brief, 2 mL blood samples from the blood collection tube with K2EDTA (BD Vacutainer^®^, Plymouth, UK) were placed into the Leucosep TM tube (Bio-check Laboratories Ltd., New Taipei City, Taiwan) to remove red blood cells from whole blood through Ficoll–Paque PLUS (GE Healthcare Life Sciences, Taipei, Taiwan) by concentration gradient. After centrifugation, there are four separated layers normally, in which the red blood cells having higher density than Ficoll are deposited below the Ficoll layer, the mononucleated cells layer containing CTC (peripheral blood mononuclear cells (PBMCs)), potential CTM, and other lymphocytes, are suspended above the Ficoll layer because of lower density than Ficoll, and the top layer above the CTC-containing layer was the plasma layer. Then, plasma and CTC containing layers were collected both for each sample to perform another centrifugation to deposit cell pellets and isolate plasma from cells and debris. After collection, plasma-containing supernatants were kept in −20 °C temporarily before exosome and miRNA extractions, and the CTC/CTM-containing pellets were collected, resuspended, and stained by Hoechst33258 (Thermo Fisher Scientific Taiwan Co., Ltd., Taipei, Taiwan), EpCAM-FITC (BioMab Inc., Taipei, Taiwan) and CD45-pecy7 (Beckman Coulter Inc, Brea, CA, USA) antibodies according to corresponding instructions, before being performed on our novel platform assisted by the patent microfluidic chip (SACA chip) to visualise and count numbers. Details about processes of CTC/CTM detection and enumerations could be found in our previous studies [14,27].

#### 2.2.2. Exosome Extraction and Identification

Exosomes were isolated by miRCURY Exosome Serum/Plasma kit (Cat no.76603) from plasma sample according to instruction. In brief, the plasma samples were passed through a 0.8 μm filter (Minisart^®^ Syringe Filter, Surfactant-free Cellulose Acetate) to exclude excessive vesicles before being mixed with 4 μL Thrombin and then incubated for 5 min at room temperature followed by centrifugation at 1000× *g* for 5 min. About 0.3 mL supernatant for each sample was collected and mixed with 120 μL Precipitation Buffer A. The mixture was then vortexed and incubated at 4 °C for 60 min. After another centrifugation at 500× *g* for 5 min, 280 μL Resuspension Buffer was added to resuspend the exosome pellet. Exosome samples were stored at −20 °C. The morphology and size of the extracted exosomes were characterised by scanning electron microscopy (HRFEG-SEM, JEOL, JSM-7610F, Japan), nanoparticle tracking analysis (NTA) and Western blot (WB) (Supplementary Data).

#### 2.2.3. miRNAs Extraction and miR-21 Gene Expression

Total RNA containing miRNAs from plasma and exosomes of every sample was extracted by QIAzol-based miRNeasy extraction kit (Qiagen) under instructions from the manufacturer. The final concentration and purity of RNAs after elution were measured by a spectrophotometer (BioTek Epoch 2 with Take3 plate) and kept in −80 °C fridge. Based on the concentration of each sample, expressions of miR-21 in every plasma sample were detected by qRT-PCR (miRCURY LNA Kit, Qiagen) with specific primer essay (Cat no.YP00204230).

In brief, 10 ng RNA template, 2 μL 5× miRCURY RT Reaction Buffer and 1 μL 10× miRCURY Enzyme Mix were gently mixed with RNase-free water to form a final volume of 10 μL system in the reaction tube and were centrifuged before being incubated at 42 °C for 60 min. Then, samples were incubated at 95 °C for 5 min to inactivate the reverse transcriptase. The resultant 10 μL cDNA solution of each sample was stored at −20 °C fridge.

Detection of the gene expression of miR-21 (from plasma or exosome) by qPCR was performed on the Mic (Magnetic Induction Cycler) PCR Machine (BMS). In brief, 3 μL 1:30 diluted cDNA template, 5 μL 2× miRCURY SYBR Green Master Mix, 1 μL miRCURY primer assay, and 1 μL RNase-free water were mixed into a final volume of 10 μL PCR reaction solution as one replica for qPCR test. Every sample was tested at least twice, and each test at least contained three replicas in each qPCR run under the condition of 2 min initial denaturation at 95 °C, followed by 40 amplification cycles in which each cycle consisted of 10 s denaturation at 95 °C, and 60 s annealing and extension at 56 °C.

### 2.3. Statistical Analysis

Relative expressions of miR-21 in plasma and exosome extracted from plasma of 10 healthy volunteers were used as healthy control group. Relative expressions of the miR-21 gene from plasma/exosome extracted from CRC patients were further quantitated by using the 2^−∆∆Ct^ ∆method [58], and miR-16 was selected to be the reference gene.

All statistical analyses including x^2^ or Fisher exact test, Mann–Whitney U test, Kruskal–Wallis test, Receiver Operating Characteristic (ROC) curve, AUC, and Kaplan–Meier survival analyses were performed by GraphPad Prism 8 (GraphPad Software Inc. San Diego, CA, USA) for Windows, and Microsoft^®^ Excel (Microsoft^®^ office 365 version 2019) was used for other calculations and tables. All *p* values were statistically analysed by two-sided test, and *p* value < 0.05 was considered statistically significant.

(1) $$\mathrm{Recurrence}\;\mathrm{rate}=\frac{\mathrm{the}\;\mathrm{number}\;\mathrm{of}\;\mathrm{recurrent}\;\mathrm{patients}}{\mathrm{the}\;\mathrm{number}\;\mathrm{of}\;\mathrm{non}-\mathrm{recurrent}\;\mathrm{patients}+\mathrm{the}\;\mathrm{number}\;\mathrm{of}\;\mathrm{recurrent}\;\mathrm{patients}}\times 100\%$$

Recurrence rate was determined by Equation (1):

Odds ratio was defined as the odds of disease in the experimental group and the odds of disease in the control group for prospective research and retrospective research. (Equation (2) takes an example of results to assess CTC cumbers.)

(2) $$\begin{array}{c} \mathrm{Odds}\;\mathrm{ratio}\;\mathrm{of}\;\mathrm{CTCs}\\ =\frac{\mathrm{the}\;\mathrm{number}\;\mathrm{of}\;\mathrm{recurrent}\;\mathrm{patients}\;\mathrm{with}\;\mathrm{high}\;\mathrm{CTC}\;\mathrm{number}\times \mathrm{the}\;\mathrm{number}\;\mathrm{of}\;\mathrm{non}-\mathrm{recurrent}\;\mathrm{patients}\;\mathrm{with}\;\mathrm{low}\;\mathrm{CTC}\;\mathrm{number}}{\mathrm{the}\;\mathrm{number}\;\mathrm{of}\;\mathrm{recurrent}\;\mathrm{patients}\;\mathrm{with}\;\mathrm{low}\;\mathrm{CTC}\;\mathrm{number}\times \mathrm{the}\;\mathrm{number}\;\mathrm{of}\;\mathrm{non}-\mathrm{recurrent}\;\mathrm{patients}\;\mathrm{with}\;\mathrm{high}\;\mathrm{CTC}\;\mathrm{number}} \end{array}$$

DFS was calculated according to dates of treatment and recurrence of patients with stage I to III CRC, while PFS was calculated according to dates of treatment and recurrence of patients with stage IV CRC.

## 3. Results

### 3.1. Demographics of Patients with CRC

There were 113 CRC cases finally enrolled in this study from 2019 to 2020 (Table 1). These peripheral blood (PB) samples were collected at Veterans General Hospital, Taipei, Taiwan, before surgical operations and were sent to NTHU to detect the number of CTCs/CTM as the previous paper mentioned [14,27], as well as to isolate the plasma for further miRNA and exosome extractions. Non-CRC patients or cases diagnosed with stage zero were excluded from this study. The average age of patients was around 65 years old in total cases, and most of them were male (68%), diagnosed with T3–T4 stage (65%), and without regional lymph node metastasis (62%) or distant metastasis (90%). Based on the Union for International Cancer Control (UICC) and American Joint Committee on Cancer (AJCC) TNM classification, there were 36 cases (32%) with stage I, 31 cases (27%) with stage II, 35 cases (31%) with stage III and 11 cases (10%) with stage IV disease. In most cases, patients did not experience any kind of adjuvant treatment such as chemotherapy or radiotherapy (84%) before first surgical operation, and their tumour sizes were smaller than 5 cm^2^ (79%) but could be differentiated moderately (93%). About three quarters of these patients were diagnosed as colon cancer, and the rest were rectal cancer (27%). Conventional serum tumour markers for CRC such as the CEA (66%) and CA19-9 (87%) in more than half of these PB samples were assessed as low level, when the thresholds were set as 5 ng/mL and 37 U/mL, respectively, in this study according to clinical suggestions [14,59]. The number of patients was also categorised by the expression levels of plasma/exosomal miR-21 and enumerations of CTC/CTM [14,27] (Table 1), in which the best decisive thresholds of each analysis of these biomarkers were selected by referencing the sensitivity and specificity of the Receiver Operating Characteristic (ROC) curves plotted to prognose the recurrent incidents of these samples. Data above their individual cut-off values were classified into the high group; otherwise, they were in the low group. *p* values of the clinicopathological data were analysed by Fisher’s exact test or Chi-squared test (GraphPad prism 8). From the results of cases classified by miR-21 (Table 1), only in the category of CA19-9 level was there a significant difference (*p* = 0.0058) in patients stratified by the expression of exo-miR-21, but in the category of the TNM stage (*p* = 0.0003), especially in N (*p* = 0.0133) and M (*p* = 0.0004) stages, and in the category of pre-operative CA19-9 level (*p* = 0.0257), were there significant differences in patients subdivided by expressions of plasma miR-21. From the results of patients classified by numbers of CTCs/CTM (Table 1), only in the M stage (*p* = 0.0422) and pre-operative serum CEA level (*p* = 0.0006) groups were cases categorised by the presence of CTM showed significant differences. In other subgroups, there were no pronounced differences observed.

### 3.2. Distributions and Correlations of Plasma/Exosomal miR-21 Expression in Stages

#### 3.2.1. Distributions of Plasma/Exosomal miR-21 Expressions

**Figure 1:**
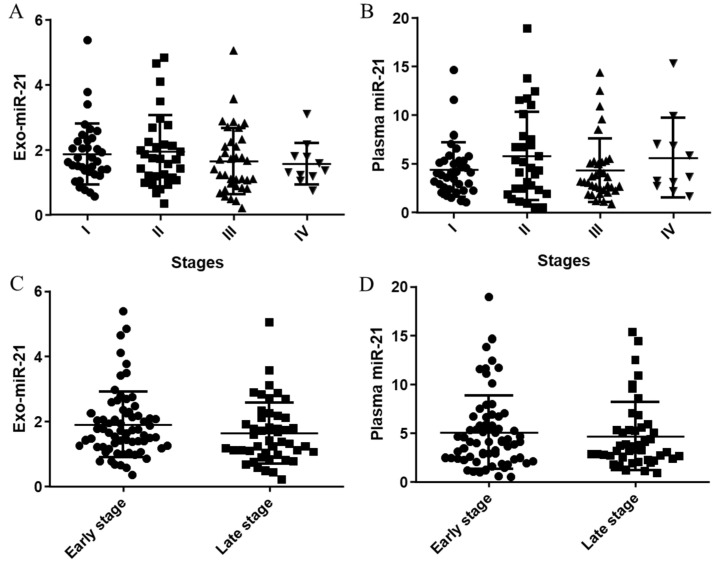
Distributions of the relative expression of plasma miR-21 and exo-miR-21 in stages. (**A**) Distributions of the relative expression of exosomal miR-21 in all stages. (**B**) Distributions of the relative expression of plasma miR-21 in all stages. (**C**) Distributions of the relative expression of exosomal miR-21 in early stage and late stage. (**D**) Distributions of the relative expression of plasma miR-21 in early stage and late stage.

The relative expression levels of plasma and exosomal miR-21 in 113 CRC patients in all (Figure 1A,B), early (stage I and II) and late stages (stage III and IV) (Figure 1C,D) are plotted in Figure 1. There were no big differences of the median expressions from both plasma and exosomal miR-21 in stages, and similarly, the median expressions of both plasma (*p* = 0.1425) and exosomal miR-21(*p* = 0.5017) in late stages were slightly lower than those in the early stages.

#### 3.2.2. Correlations between Plasma miR-21 and exo-miR-21 Expressions

**Figure 2:**
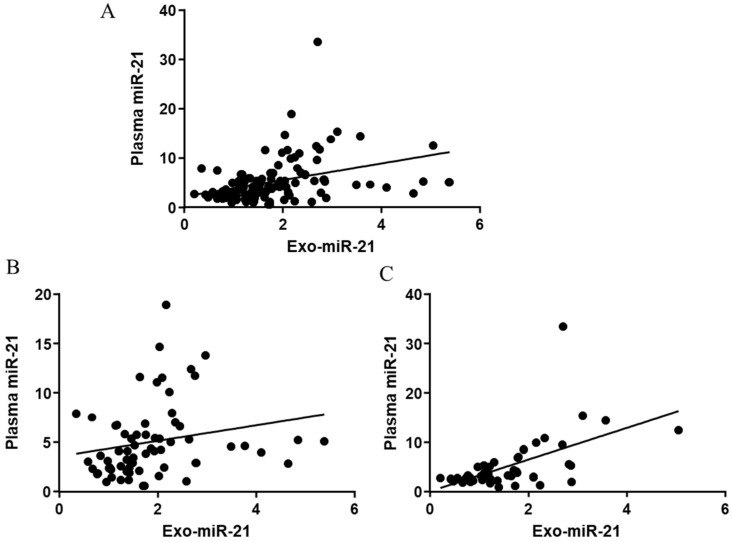
Correlations between plasma miR-21 and corresponding exo-miR-21 in stages. Correlations between plasma miR-21 and exosomal miR-21 in all stages (**A**), early stage (**B**) and late stage (**C**).

To explore the relationship between the miR-21 extracted from plasma and plasma-derived exosomes, correlations between plasma miR-21 and exo-miR-21 in all, early and late stages were investigated (Figure 2). Except for a positive trend (Pearson r = 0.2142, *p* = 0.0841) between expressions of plasma miR-21 and exo-miR-21 in the early stage (Figure 2B), significantly positive correlations (Figure 2A,C) between plasma miR-21 and exo-miR-21 in all stages (Pearson r = 0.3643, *p* < 0.0001) and in late stages (Pearson r = 0.5554, *p* < 0.0001) were observed, suggesting a close relationship between plasma miR-21 and exo-miR-21.

### 3.3. Correlations between Plasma/Exosomal miR-21 Expressions and Other Biomarkers

#### 3.3.1. Correlations between Plasma/Exosomal miR-21 Expressions and Enumerations of EpCAM Positive CTCs (EpCTCs)

**Figure 3:**
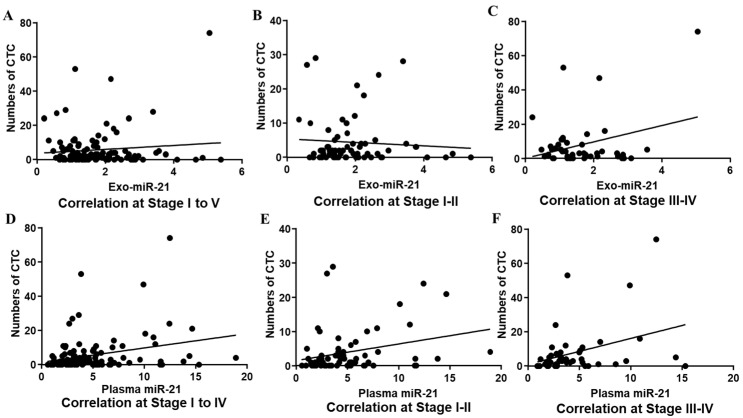
Correlations between plasma/exosomal miR-21 and PB EpCTC numbers in stages. Correlations between exosomal miR-21 and numbers of CTC in all stages (**A**), early stage (**B**) and late stage (**C**). Correlations between plasma miR-21 and numbers of CTC in all stages (**D**), early stage (**E**) and late stage (**F**).

The correlativity between plasma/exosomal miR-21 expressions and numbers of EpCAM positive CTC detected in the identical PB samples are plotted in Figure 3. The results indicate that there was a significant positive correlation between CTCs numbers and exo-miR-21 levels in late stages (Figure 3C: Pearson r = 0.3023, *p* = 0.0436) but not in the early stages (Figure 3B: Pearson r = −0.0.072, *p* = 0.5624) and all stages (Figure 3A: Pearson r = 0.1045, *p* = 0.273). In contrast, the expression levels of plasma miR-21 presented significant associations with the number of CTCs (Figure 3D–F) in all stages (Figure 3D: Pearson r = 0.278, *p* = 0.0033) and both early (Figure 3E: Pearson r = 0.2776, *p* = 0.024) and late stages (Figure 3F: Pearson r = 0.3525, *p* = 0.0189).

#### 3.3.2. Correlations between Plasma/Exosomal miR-21 Expressions and Other Biomarkers

Correlations between plasma/exosomal miR-21 expressions and pre-operative serum CEA and CA19-9 levels are plotted in the Supplementary Data (Figures S1 and S2). From these results, there were no significant correlations between plasma (Figure S1D–F)/exosomal miR-21 (Figure S1A–C) and CEA levels in all stages (Figure S1A: Pearson r = −0.0251, *p* = 0.8025; D: Pearson r = 0.0272, *p* = 0.7881), and both early (Figure S1B: Pearson r = 0.1207, *p* = 0.3345; E: Pearson r = 0.1139, *p* = 0.3664) and late stages (Figure S1C: Pearson r = −0.1727, *p* = 0.3139; F: Pearson r = −0.0625, *p* = 0.7216); but significant correlations between both plasma (Figure S2D,E)/exosomal (Figure S2A,B) miR-21 and CA19-9 level in all stages (Figure S2A: Pearson r = 0.3377, *p* = 0.0005; D: Pearson r = 0.2712, *p* = 0.0063) and early stages (Figure S2B: Pearson r = 0.3747, *p* = 0.0019; E: Pearson r = 0.2935, *p* = 0.0177), rather than in late stages (Figure S2C: Pearson r = 0.3169, *p* = 0.0597; F: Pearson r = 0.2754, *p* = 0.1903), were observed.

### 3.4. Prediction of CRC Recurrence in Patients Stratified by Biomarkers Individually and Combined

#### 3.4.1. Receiver Operating Characteristic (ROC) Curves and Kaplan–Meier Survival Analyses of Plasma/exo-miR-21 Individually to Predict CRC Recurrence in Patients

**Figure 4:**
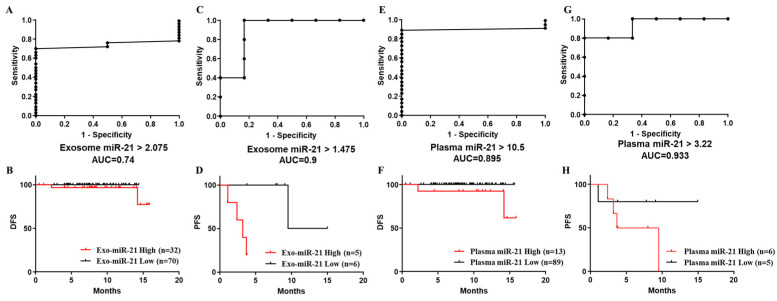
Receiver Operating Characteristics (ROC) curve and Kaplan–Meier survival analyses of plasma/exo-miR-21 individually to prognose CRC recurrence. (**A**) ROC and Area Under ROC Curve (AUC) in patients with stage I to III CRC, stratified by the selected cut-off points of exo-miR-21. (**B**) Disease-free survival (DFS) curve by the Kaplan–Meier survival analysis to discriminate recurrence in patients with stage I to III CRC, stratified by cut-off pointes of exo-miR-21. (**C**) ROC and AUC in patients with stage IV CRC, stratified by the selected cut-off points of exo-miR-21. (**D**) Progression-free survival (PFS) curve by the Kaplan–Meier survival analysis to discriminate recurrence in patients with stage IV CRC, stratified by cut-off pointes of exo-miR-21. (**E**) ROC and AUC in patients with stage I to III CRC, stratified by the selected cut-off points of plasma miR-21. (**F**) DFS curve by the Kaplan–Meier survival analysis to discriminate recurrence in patients with stage I to III CRC, stratified by cut-off pointes of plasma miR-21. (**G**) ROC and AUC in patients with stage IV CRC, stratified by the selected cut-off points of plasma miR-21. (**H**) PFS curve by the Kaplan–Meier survival analysis to discriminate recurrence in patients with stage IV CRC, stratified by cut-off pointes of plasma miR-21.

Different cut-off points (stage I to III and stage IV) were selected for individual biomarkers, and ROC curves of exo-miR-21 (Figure 4A,C), plasma miR-21 (Figure 4E,G) and the corresponding Kaplan–Meier survival analyses (DFS and PFS) (Figure 4B,D,F,H respectively) are presented in Figure 4. Considering the selected cut-off points, the Area Under ROC Curves (AUC) in plots were all dropped in the range between 0.7 and 0.9, showing relatively higher accuracies. Compared to the prediction in patients with stage I to III CRC presented by the DFS curve of exo-miR-21 (Figure 4B: *p* = 0.1423, Hazard Ratio (HR) was undefined at 95% CI), recurrent patients with stage IV CRC could be discriminated significantly in the PFS curve (Figure 4D: *p* = 0.0068, HR = 0.909, 95% CI = 1.429–68.72) of exo-miR-21, whereas in the survival analyses of patients stratified by expression levels of plasma miR-21, recurrent patients could be both discriminated in DFS and PFS curves (Figure 4H: *p* = 0.2424, HR = 3.405, 95% CI = 0.5865–19.76), especially in DFS with significance (Figure 4F: *p* = 0.023, HR was undefined at 95% CI.). 

#### 3.4.2. Kaplan–Meier Survival Analyses on DFS of a Series of Biomarkers Individually and Combined to Predict Recurrence in Patients with Stage I to III CRC

**Figure 5:**
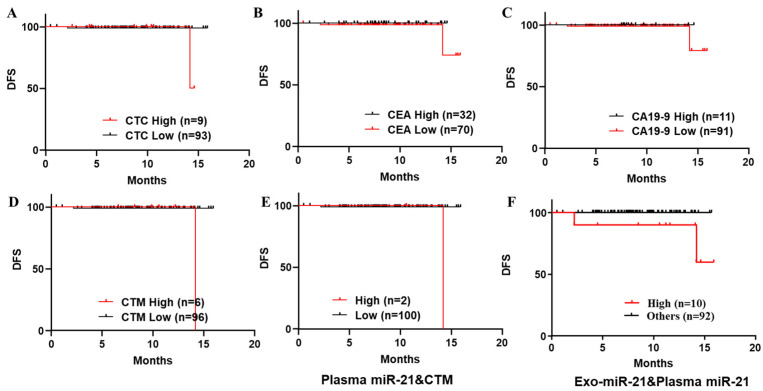
Kaplan–Meier survival analyses on DFS curves to differentiate recurrence in patients with stage I to III CRC by individual and combined biomarkers. (**A**–**D**) DFS curve to differentiate recurrence in patients with stage I to III CRC stratified by numbers of CTC (**A**), serum CEA level (**B**), CA19-9 (**C**) and presence of CTM (**D**), individually. (**E**) DFS curve to differentiate recurrence in patients with stage I to III CRC stratified by considering the combination of plasma miR-21 and CTM. (**F**) DFS curve to differentiate recurrence in patients with stage I to III CRC stratified by considering the combination of plasma and exosomal miR-21.

Different cut-off points (stage I to III and stage IV) were selected for a series of biomarkers individually, and ROC curves and the AUC are presented in Figure S3 (Supplementary data). Based on the selected cut-off points of every biomarker examined in this study (Figure 4 and Figure S3), including plasma/exosomal miR-21, EpCTCs/CTM and pre-operative serum CEA/CA19-9, the Kaplan–Meier survival analyses on DFS of these biomarkers individually and combined (Figure 5 and Figure S4) to predict recurrence in patients with stage I to III CRC were investigated. From these results, there was no significance (Figure 5A: *p* = 0.2957, HR = 3.724, 95% CI = 0.1252–110.8; B *p* = 0.33, HR = 0, 95% CI = −1.000–1.000; C: *p* = 0.5698, HR = 0, 95% CI = −1.000–1.000; D: *p* = 0.0801,HR = 7.824, 95% CI = 0.09878–619.7) in predicting the CRC recurrence in stage I to III patients subdivided by these biomarkers examined individually, although patients with a high level of most markers had worse survival outcomes. The cut-off points of pre-operative serum CEA/CA19-9 levels were selected by clinical suggestion rather than by calculations, and their AUCs were dropped in the range of 0.5 to 0.8 (Figure S3). The results of the Kaplan–Meier survival analyses on DFS-curves-stratified patients with low levels of CEA/CA19-9 presented worse outcomes (Figure 5B,C).

However, in the DFS curves of patients stratified by those biomarkers individually and combined (Figure 5 and Figure S4), there was no significance observed in most results (Figure S4), except for those considering both plasma miR-21 and CTM (Figure 5E: *p* = 0.0411, HR = 9.714, 95% CI = 0.08285–1139), or both plasma miR-21 and exo-miR-21 (Figure 5F: *p* = 0.0164, HR was undefined at 95% CI).

#### 3.4.3. Kaplan–Meier Survival Analyses on PFS of a Series of Biomarkers Individually and Combined to Predict Recurrence in Patients with Stage IV CRC

**Figure 6:**
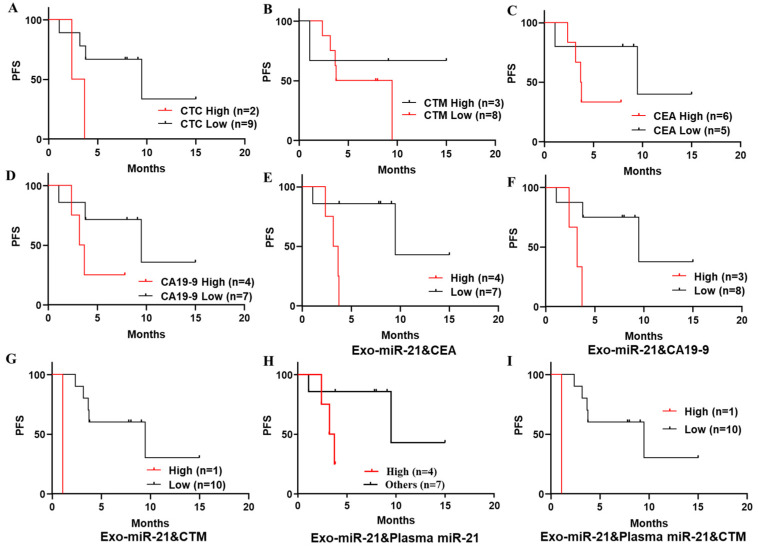
Kaplan–Meier survival analyses on PFS curves to differentiate recurrence in patients with stage IV CRC by individual and combined biomarkers. (**A**–**D**) PFS curves to differentiate recurrence in patients with stage IV CRC stratified by numbers of CTC (**A**), presence of CTM (**B**), serum CEA (**C**), and CA19-9 levels (**D**). (**E**–**I**) PFS curves to differentiate recurrence in patients with stage IV CRC stratified by combined biomarkers.

Based on the selected cut-off point of every biomarker examined in this study (Figure 4 and Figure S3), Kaplan–Meier survival analyses on PFS curves to predict CRC recurrence in patients stratified by several biomarkers individually and combined (Figure 6 and Figure S5) were studied. From these results, there was no significance (Figure 6A: *p* = 0.0546, HR = 4.355, 95% CI = 0.3131–60.58; B: *p* = 0.499, HR = 0.4934, 95% CI = 0.08436–2.886; C: *p* = 0.2165, HR = 2.563, 95% CI = 0.5110–12.86; D: *p* = 0.1317, HR = 3.027, 95% CI = 0.4750–19.29) observed in predicting recurrence in patients with stage IV CRC subdivided by the examined biomarkers (numbers of CTC, presence of CTM, CEA and CA19-9 levels), individually. Although, in most results, patients with higher level of biomarkers (Figure 6A,C,D), presented visibly worse outcomes.

By considering combined biomarkers, based on the significance found in individual study (Figure 4 and Figure 5), some valuable results of PFS curves are plotted in Figure 6E–I, and others are collected in the Supplementary Data (Figure S5). Significant results were observed in PFS curves of patients stratified by exo-miR-21 combined with serum CEA (Figure 6E: *p* = 0.0122, HR = 6.055, 95% CI = 0.9500–38.59), CA19–9 (Figure 6F: *p* = 0.0122, HR = 5.522, 95% CI = 0.5993–50.89), or CTM (Figure 6G: *p*= 0.0016, HR = 13, 95% CI = 0.01858–9094), and by plasma miR-21 combined with CTM (Figure 6I: *p*= 0.0016, HR = 13, 95% CI = 0.01858–9094), whereas patients with stage IV CRC stratified by a high level of both plasma and exosomal miR-21s (Figure 6H: *p* = 0.0782, HR = 4.068, 95% CI = 0.5641–29.34) presented worse survival outcomes without significance.

#### 3.4.4. Recurrence Rate and Odds Ratio in Predicting CRC Recurrence in Patients in All Stages and Late Stages

##### Recurrence Rate and Odds Ratio in CRC Patients in ALL Stages

The recurrence rate and odds ratio in predicting CRC recurrence in 113 patients in all stages were assessed, based on individual and combined biomarkers (Table 2). For individual biomarkers, compared to patients grouped by low-level biomarkers, corresponding patients with high-level markers showed high recurrence rates such as 18.9% in high-level exo-miR-21 group, 37% in high-level plasma miR-21 group, 27.3% in high CTCs group, 22.2% in high CTM group, 10.5% in high CEA group, and 20% in high CA19-9 group. The odds ratios (ORs) of exo-miR-21, plasma miR-21 and CTCs were 17.5 (*p* = 0.0016), 54.3 (*p* < 0.0001) and 7.3 (*p* = 0.0298), much higher than those of CTM, serum CEA and CA19-9 groups, which were 4.7 (*p* = 0.123), 2.1 (*p* = 0.4388) and 4.7 (*p* = 0.071), respectively.

Compared to those of individual biomarkers, high recurrence rates and significant ORs were found (Table 2) in the results considering combined biomarkers, such as 42.9% recurrence rate (ORs = 36.4, *p* < 0.0001) in the group considering both plasma and exosomal miR-21, 50% in the group considering plasma/exosomal miR-21 together with CTC (ORs = 20.4, *p* < 0.0043) or with CTM (ORs = 14.9, *p* < 0.1372), 44.4% in the group considering plasma/exosomal miR-21 together with CEA (ORs = 20, *p* < 0.0012), and 50% in the group considering plasma/exosomal miR-21 together with CA19-9 (ORs = 20.4, *p* < 0.0043).

##### Recurrence Rate and Odds Ratio in CRC Patients in Late Stages

The recurrence rate and odds ratio to predict the recurrence in CRC patients in late stages were also investigated, based on individual and combined biomarkers (Table S1). For individual biomarkers, similarly, compared to patients grouped by low-level biomarkers, corresponding patients with high-level markers showed high recurrence rates, including 37.5% in high exo-miR-21 group, 60% in high plasma miR-21 group, 50% in high CTCs group, 40% in high CTM group, 16.7% in high CEA group, and 30% in high CA19-9 group. In addition, significant ORs were found in stage IV patients stratified by exo-miR-21, plasma miR-21 and CTCs, which were 17.4 (*p* = 0.0047), 52.5 (*p* = 0.0001) and 9 (*p* = 0.037) respectively, higher than those found in patients grouped by CTM, serum CEA and CA19-9, which were 4.8 (*p* = 0.1599), 1.3 (*p* = 1.0) and 3.4 (0.1632), respectively. 

High recurrence rates and significant ORs were also observed in results considering combined biomarkers, compared to those analysed individually, such as 62.5% (ORs = 30, *p* = 0.0008) in the group considering both plasma and exosomal miR-21s, 75% in the group considering the plasma/exosomal miR-21s together with CTC (ORs = 28.5, *p* = 0.0086), 66.7% in the group considering the plasma/exosomal miR-21s together with CEA (ORs = 24.7, *p* = 0.0029), and 60% in the group considering the plasma/exosomal miR-21s together with CA19-9 (ORs = 13.9, *p* = 0.0199). Although a 100% recurrence rate was found in group considering the plasma/exosomal miR-21 together with CTM, there was no OR due to the rare cases found in these samples.

## 4. Discussion and Conclusions

Plasma/exosomal microRNAs, CTCs/CTM and serum CEA/CA19-9 are all non-invasive promising biomarkers with highlighted clinical values in CRC [8,25,26,30,41,42,53,60], but as individual biomarkers, they have different sensitivity and specificity, leading to the hypothesis and suggestions by a few studies that the diagnostic and prognostic values of individual biomarkers could be improved by additional biomarkers, and better outcomes of diagnosing and prognosing the recurrence and metastasis of CRC would be offered by analysing combined biomarkers. For example, increases in preoperative serum markers in cancer patients before operation and relapse were both observed in CRC cases [60], whereas improved prognostic values in predicting survivals and in monitoring recurrence and metastasis in CRC patients were reported by studying CEA and CA19-9 levels together [54] or by analysing the combination of CEA, CA19-9 with CA242 [55]. Significant correlations between CTC numbers and two other biomarkers in blood (CEA and the platelet-to-lymphocyte ratio (PLR)) in recurrent patients with high-risk stage II or stage III CRC, and between the high number of CTCs (cut-off point was 20 cells/8 mL blood) and the presence of CTM in patients, were confirmed by Abdallah EA et al. [21]. A recent study by Chiu SY et al. revealed the diagnostic value of CTC enumerations and found that improved accuracies of AUC in predicting metastatic CRC samples were obtained by analysing the combination of CEA and CTCs, rather than by CEA alone [56]. We previously studied the number of CTCs, presence of CTM, levels of CEA and CA19-9 in 163 PB samples in all CRC stages and uncovered that compared to using single markers such as the CTC number or CEA level, higher odds ratio in predicting CRC recurrence in patients could be acquired by analysing the combination of CTCs and CEA, and significantly higher recurrence rates were observed in patients grouped by a panel of biomarkers [14]. Improved prognostic values of the combination of plasma miRNAs with serum markers in CRC patients in the follow-up have been reported by Pesta M et al. [57], in which best survival outcomes after surgery in CRC patients were predicted by analysing the combination of a panel of highly sensitive miRNAs (miR20, miR20a and miR-23a) with specific serum markers CEA/CA19-9.

In this paper, expressions of miR-21 in plasma and plasma-derived exosomes were both evaluated by qPCR. Through a novel platform and our patent self-assembled cell array chip (SACA) developed in previous studies [14,27], the number of the EpCAM-positive CTC and the presence of CTM in samples were also visualised. In combination with information on serum markers, the potential to predict CRC recurrence in patients stratified by several valuable CRC biomarkers individually and combined, at an early time of follow-up after their first surgical treatment were successfully assessed. Since no big difference between distributions of the circulating miR-21s in different TNM stages (Figure 1) and since significant correlations between expressions of plasma and exosomal miR-21 in all stages and late stages (Figure 2) were found, a close relationship between plasma miR-21 and exo-miR-21 with different sensitivity and specificity, regarding their origins, could be suggested, which could be confirmed by the following results, where similar correlations of the plasma/exosomal miR-21s with serum CEA (Figure S2) and CA19-9 levels (Figure S3), and a slight difference in their correlations with CTCs (Figure 3) were observed.

Separated cut-off points, resulting from the selection of best sensitivity and specificity of individual biomarkers, were achieved for stratifying patients in analyses. MiR-21s from both plasma and exosomes presented high accuracies to predict CRC recurrence in stratified patients, in which stage IV CRC patients with high levels of exo-miR-21 and CRC patients in stage I to III with high levels of plasma miR-21 had significantly worse survival outcomes. In the results of individual biomarker analysis in predicting CRC recurrence in patients in all stages and late stages, a higher recurrence rate and odds ratio with significances were observed in patients grouped by plasma/exosomal miR-21s, and CTCs, versus those grouped by CTM, CEA/CA19-9, individually. Comparing to results of individual analysis, a higher recurrence rate and improved odds ratios could be obtained by investigating combinations of plasma and exosomal miR-21s, and by analysing plasma/exosomal miR-21s together with other biomarkers, confirming previous results and demonstrating the potential to improve the prediction and monitoring on CRC recurrence in patients (especially in late stages) at early time of follow-up after their treatments, by analysing several biomarkers together, especially containing the circulating miR-21s, with CTCs/CTM or with serum markers CEA/CA19-9.

Although our results need to be further validated in future studies, the number of samples, especially the advanced cases and recurrent cases in this study, were limited due to samples selected at early follow-up visits. Here, we want to conclude that the CTCs/CTM in our blood samples of CRC patients as sensitive biomarkers were comparable to serum markers, and even better performed in predicting recurrence in late-stage CRC patients. The circulating miR-21s have highly potential values to predict early recurrence in CRC patients. Furthermore, improved prognosing efficacy in CRC recurrence and better outcomes to significantly differentiate recurrence in stratified patients could be obtained by analysing combined biomarkers, rather than by examining individual one.

## Figures and Tables

**Table 1:** Preoperative demographics, classifications, and pathological parameters of 113 CRC patients (* *p* < 0.05; ** *p* < 0.01; *** *p* < 0.001).

		Exosome miR21 in PB			Plasma miR21 in PB			CTCs in PB			CTM in PB		
	Total	High	Low	*p*	High	Low	*p*	High	Low	*p*	High	Low	*p*
	N = 113	N = 37	N = 76		N =20	N = 93		N = 11	N = 102		N = 9	N = 104	
Age	65.19	67.08	64.28		66.5	64.91		65.19	69.45			61.22	65.54
	(39–93)	(42–93)	(39–90)		(42–93)	(39–92)		(49–85)	(39–92)			(40–83)	(39–93)
Gender													
Male	77 (68%)	26	51	0.8311	13	64	0.7936	6	71	0.3231	6	71	>0.9999
Female	36 (32%)	11	25		7	29		5	31		3	33	
TNM Stage													
I	36 (32%)	10	26	0.7189	3	33	0.0003 ***	3	33		3	33	0.0788
II	31 (27%)	11	20		6	25		2	29	0.6896	1	30	
III	35 (31%)	11	24		4	31		4	31		2	33	
IV	11 (10%)	5	6		7	4		2	9		3	8	
T stage													
T1–T2	39 (35%)	10	29	0.2947	3	36	0.0674	3	36	0.7454	3	36	>0.9999
T3–T4	74 (65%)	27	47		17	57		8	66		6	68	
N stage													
N0	70 (62%)	23	47	0.4572	11	59	0.0133 *	6	64	0.2162	6	64	0.9487
N1	30 (27%)	8	22		3	27		2	28		2	28	
N2	13 (12%)	6	7		6	7		3	10		1	12	
M stage													
M0	102 (90%)	32	70	0.4996	13	89	0.0004 ***	9	93	0.2905	6	96	0.0422 *
M1	11 (10%)	5	6		7	4		2	9		3	8	
Tumor size (cm^2^)													
≥5	24 (21%)	12	12	0.0521	7	17	0.1302	5	19	0.0537	2	22	>0.9999
<5	89 (79%)	25	64		13	76		6	83		7	82	
Differentiation													
Poor	5 (4%)	1	4	0.8246	1	4	0.7619	1	4	0.2641	1	4	0.1449
Moderate	105 (93%)	35	70		18	87		9	96		7	98	
Well	3 (3%)	1	2		1	2		1	2		1	2	
Location													
Right colon	26 (23%)	10	16	0.7366	4	22	0.3203	4	22	0.0781	2	24	0.8861
Left colon	57 (50%)	17	40		8	49		2	55		4	53	
Rectal	30 (27%)	10	20		8	22		5	25		3	27	
CEA (5 ng/mL)													
>5	38 (34%)	15	23	0.2958	9	29	0.2977	5	33	0.5032	8	30	0.0006 ***
≤5	75 (66%)	22	53		11	64		6	69		1	74	
CA19-9 (U/mL)													
>37	15 (13%)	10	5	0.0058 **	6	9	0.0257 *	3	11	0.1371	1	13	>0.9999
≤37	98 (87%)	27	71		14	84		8	91		8	91	
Treatments													
pre-operation	18 (16%)	6	12	>0.9999	5	13	0.3086	2	16	0.6871	2	16	0.6335
non-pre operation	95 (84%)	31	64		15	80		9	86		7	88	

**Table 2:** Recurrence rate and odds ratios (ORs) of several biomarkers individually and combined to predict CRC recurrence in 113 patients in all stages (* *p* < 0.05; ** *p* < 0.01; **** *p* < 0.0001).

All Stages	Number of Cases		Recurrence Rate (%)	Odds Ratio
113 Cases	Recurrence (+)	Recurrence (−)		
High Exosome miR-21	7	30	18.9	17.5
Low Exosome miR-21	1	75	1.3	*p* value = 0.0016 **
High Plasma miR-21	7	12	37	54.3
Low Plasma miR-21	1	93	1.1	*p* value < 0.0001 ****
High CTC	3	8	27.3	7.3
Low CTC	5	97	4.9	*p* value = 0.0298 *
Presence of CTM	2	7	22.2	4.7
Absence of CTM	6	98	5.8	*p* value = 0.123
CEA > 5 ng/mL	4	34	10.5	2.1
CEA ≤ 5 ng/mL	4	71	5.3	*p* value = 0.4388
CA19-9 > 37 U/mL	3	12	20	4.7
CA19-9 ≤ 37 U/mL	5	93	5.1	*p* value = 0.071
High Exosome miR-21	6	8	42.9	36.4 *p* value < 0.0001 ****
High Plasma miR-21				
Others	2	97	2	
High Exosome miR-21	3	3	50	20.4 *p* value = 0.0043 **
High Plasma miR-21				
High CTC				
Others	5	102	4.7	
High Exosome miR-21	1	1	50	14.9 *p* value = 0.1372
High Plasma miR-21				
With CTM				
Others	7	104	6.3	
High Exosome miR-21	4	5	44.4	20 *p* value = 0.0012 **
High Plasma miR-21				
CEA > 5 ng/mL				
Others	4	100	3.8	
High Exosome miR-21	3	3	50	20.4 *p* value = 0.0043 **
High Plasma miR-21				
CA19-9 > 37 U/mL				
Others	5	102	4.7	

## Data Availability

Data for the cohort study are available on reasonable request due to restrictions of privacy.

## Supplementary Materials

The following are available online at https://www.mdpi.com/article/10.3390/jcm11092400/s1, Figure S1: Correlations between plasma/exosomal miR-21 and serum CEA levels in stages, Figure S2: Correlations between plasma/exosomal miR-21 and serum CA19-9 levels in stages, Figure S3: Receiver Operating Characteristics (ROC) curve and Area Under ROC Curve (AUC) to differentiate recurrence in patients with stage I to III and stage IV CRC, stratified by separated cut-off points of exo-miR-21, plasma miR-21, numbers of CTC, presence of CTM and serum CEA/CA19-9 levels,, Figure S4: Disease-free survival (DFS) curves to differentiate recurrence in stratified patients with stage I to III CRC by combined biomarkers, Figure S5: Progression-free survival (PFS) curves to differentiate recurrence in stratified patients with stage IV CRC by combined biomarkers, Figure S6: Characterisations of circulating exosomes extracted from plasma, Table S1: Recurrence rate and odds ratios (ORs) to predict CRC recurrence in late-stage patients stratified by of exo-miR-21, plasma miR-21, CTCs, CTM, CEA and CA19-9 individually and combined. Supplementary methods including characterization and quantification of exosomes by scanning electron microscope (SEM), nanoparticle tracking analysis (NTA) and Western blot (WB) are described.
