# Supplementary material for: Potential Values of Circulating microRNA-21 to Predict Early Recurrence in Patients with Colorectal Cancer after Treatments
Source: J Clin Med. 2022 Apr 25;11(9):2400. doi: 10.3390/jcm11092400 (PMC9100254; doi:10.3390/jcm11092400)
Supplement: Supplementary file 1 [file jcm-11-02400-s001.zip › jcm-1652408-supplementary.pdf]

Supplementary materials: Potential values of circulating microRNA-21 to predict early recurrences in patients with colorectal cancer after treatments

Yun-Jie Hao, Chih-Yung Yang, Ming-Hsien Chen, Lu-Wey Chang, Chien-Ping Lin, Liang-Chuan Lo, Sheng-Chieh Huang, You-You Lyu, Jeng-Kai Jiang, Fan-Gang Tseng

Supplementary Figures:

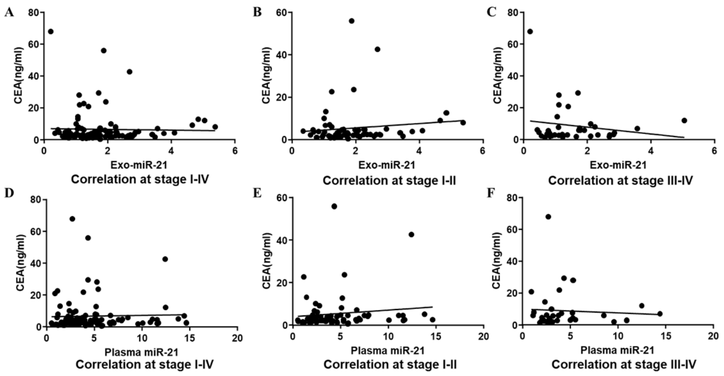

Figure S1: Correlations between plasma/exosomal miR-21 and serum CEA levels in stages

Correlations between exo-miR-21 and CEA in all stage (A, Pearson r=-0.0251, p=0.8025, N=113), in early stage (B, Pearson r=0.1207, p=0.3345, N=67), and in late stage (C, Pearson r=-0.1727, p=0.3139, N=46); Correlations between plasma miR-21 and CEA in all stage (D, Pearson r=0.0272, p=0.7881, N=113), in early stage (E, Pearson r=0.1139, p=0.3664, N=67), and in late stage (F, Pearson r=-0.0625, p=0.7216, N=46).

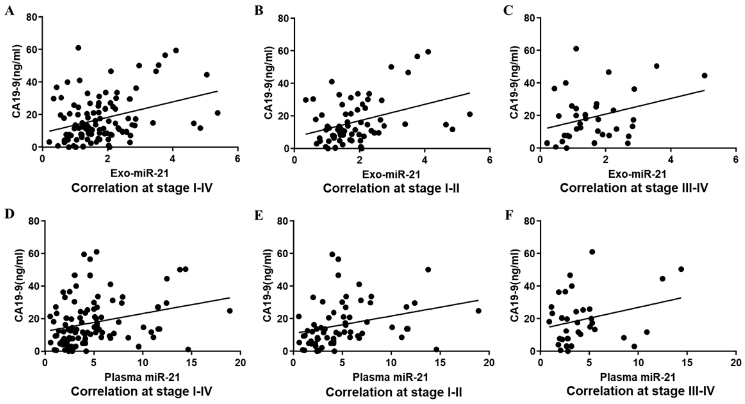

Figure S2: Correlations between plasma/exosomal miR-21 and serum CA19-9 levels in stages

Correlations between exo-miR-21 and CA19-9 in all stage (A, Pearson r=0.3377, p=0.0005, N=113), in early stage (B, Pearson r=0.3747, p=0.0019, N=67), and in late stage (C, Pearson r=0.3169, p=0.0597, N=46); Correlations between plasma miR-21 and CA19-9 in all stage (D, Pearson r=0.2712, p=0.0063, N=113), in early stage (E, Pearson r=0.2935, p=0.0177, N=67), and in late stage (F, Pearson r=0.2754, p=0.1093, N=46).

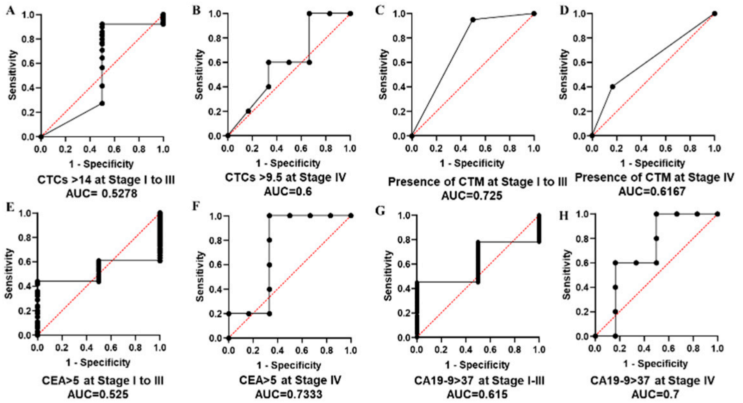

Figure S3: Receiver Operating Characteristics (ROC) curve and Area Under ROC Curve (AUC) to differentiate recurrences in patients with stage I to III and stage IV CRC, stratified by separated cut-off points of exo-miR-21, plasma miR-21, numbers of CTC, presence of CTM and serum CEA/CA19-9 levels, respectively

A: ROC curve and AUC of CRC patients in stage I to III stratified by CTC number (N=102); B ROC curve and AUC of CRC patients in stage IV stratified by CTC number (N=11); C: ROC curve and AUC of CRC patients in stage I to III stratified by the presence of CTM(N=102); D: ROC curve and AUC of CRC patients in stage IV stratified by the presence of CTM (N=11); E: ROC curve and AUC of patients in stage I to III stratified by CEA level(N=102); F: ROC curve and AUC of patients in stage IV stratified by CEA level (N=11); G: ROC curve and AUC of patients in stage I to III stratified by CA19-9 level (N=102); H: ROC curve and AUC of patients in stage IV stratified by CA19-9 level (N=11).

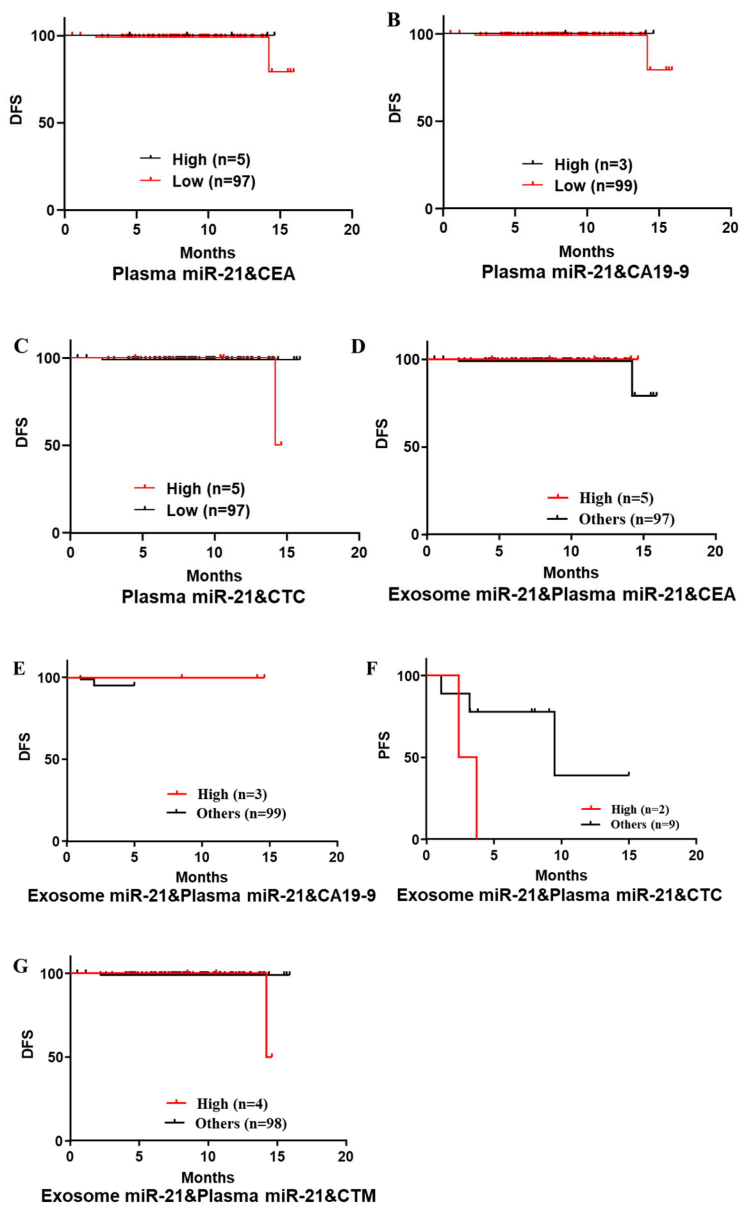

Figure S4: Disease-free survival (DFS) curves to differentiate recurrences in stratified patients with stage I to III CRC by combined biomarkers

A: DFS to differentiate recurrences in patients with stage I to III CRC stratified by plasma miR-21 combined with CEA, Hazard Ratio (HR)= 0.000, 95%CI= -1.000 to -1.000; B: DFS to differentiate recurrences in stratified patients with stage I to III CRC by plasma miR-21 combined with CA19-9, HR=0.000, 95%CI=-1.000 to -1.000; C: DFS to differentiate recurrences in stratified patients with stage I to III CRC by plasma miR-21 combined with CTCs, HR= 4.217, 95%CI= 0.1247 to 142.6; D: DFS to differentiate recurrences in stratified patients with stage I to III CRC by plasma/exosomal miR-21 combined with CEA, HR=0.000, 95%CI=-1.000 to -1.000; E: DFS to differentiate recurrences in stratified patients with stage I to III CRC by plasma/exosomal miR-21 combined with CA19-9,HR=0.000, 95%CI=-1.000 to -1.000; F: DFS to differentiate recurrences in stratified patients with stage I to III CRC by plasma/exosomal miR-21 combined with CTCs, HR=4.085, 95%CI=0.1250 to 133.5; G: DFS to differentiate recurrences in stratified

patients with stage I to III CRC by plasma/exosomal miR-21 combined with CTM, HR=4.357, 95%CI=0.1243 to 152.7.

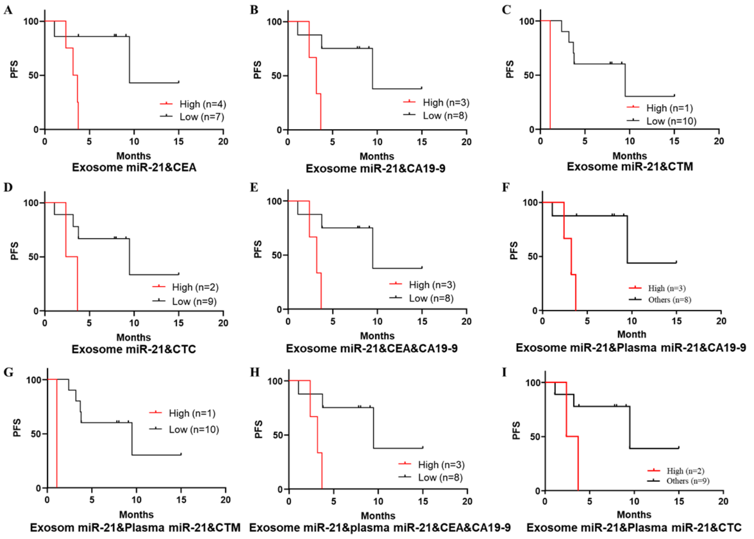

Figure S5: Progression-free survival (PFS) curves to differentiate recurrences in stratified patients with stage IV CRC by combined biomarkers

A: PFS to differentiate recurrences in stratified patients with stage IV CRC by exosomal miR-21 combined with CEA, HR= 6.055, 95%CI=0.9500 to 38.59; B: PFS to differentiate recurrences in stratified patients with stage IV CRC by exosomal miR-21 combined with CA19-9, HR= 5.522 95%CI=0.5993 to 50.89; C: PFS to differentiate recurrences in stratified patients with stage IV CRC by exosomal miR-21 combined with CTM, HR=13.00, 95%CI=0.01858 to 9094; D: PFS to differentiate recurrences in stratified patients with stage IV CRC by exosomal miR-21 combined with CTCs, HR=4.355, 95%CI=0.3131 to 60.58; E: PFS to differentiate recurrences in stratified patients with stage IV CRC by combined biomarkers of exosomal miR-21, CEA and CA19-9, HR=5.522, 95%CI=0.5993 to 50.89; F: PFS to differentiate recurrences in stratified patients with stage IV CRC by plasma/exosomal miR-21 combined with CA19-9, HR=6.653, 95%CI=0.6927 to 63.89; G: PFS to differentiate recurrences in stratified patients with stage IV CRC by plasma/exosomal miR-21 combined with CTM, HR=13.00, 95%CI=0.01858 to 9094; H: PFS to differentiate recurrences in stratified patients with stage IV CRC by combined biomarkers of plasma/exosomal miR-21, CEA and CA19-9, HR= 4.068, 95%CI=0.5641 to 29.34; I: PFS to differentiate recurrences in patients with stage IV CRC by plasma/exosomal miR-21 combined with CTC, HR= 4.728, 95%CI=0.3296 to 67.82.

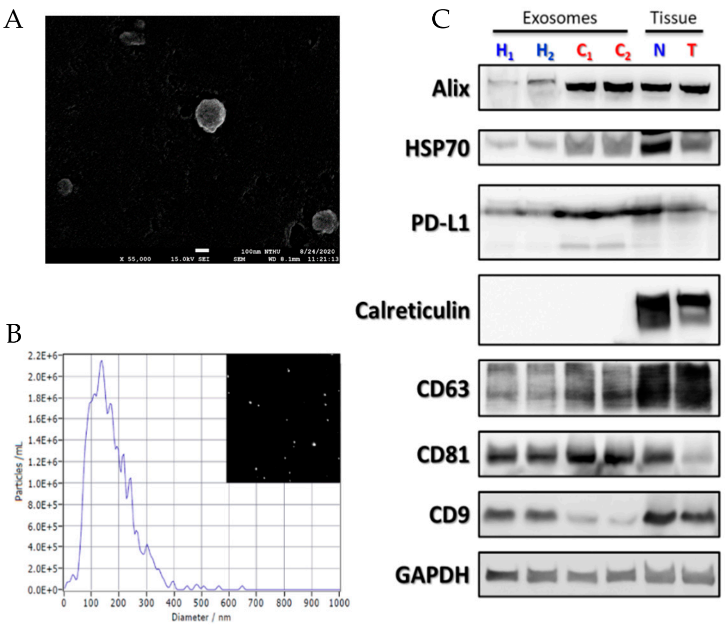

Figure S6: Characterisations of circulating exosomes extracted from plasma

A: Image of exosome by Scanning electron microscopy; B: Analysis of exosome by Nanoparticle tracking analysis; C: Analysis of exosome by western blot. H: Sample from health volunteer; C: Sample from cancer patient; N: sample from normal tissue; T: sample from tumour tissue.

Supplementary Table S1: Recurrence rate and odds ratios (ORs) to predict CRC recurrence in late-stage patients stratified by of exo-miR-21, plasma miR-21, CTCs, CTM, CEA and CA19-9 individually and combined. (*, p<0.05; **, p<0.01; ***, p<0.001)

Late Stages 46 cases	Number of cases		Recurrence rate (%)	Odds Ratio
	Recurrence(+)	Recurrence(-)		
High Exosome miR-21	6	10	37.5	17.4
Low Exosome miR-21	1	29	3.3	p value=0.0047**
High Plasma miR-21	6	4	60	52.5
Low Plasma miR-21	1	35	2.8	p value=0.0001***
High CTC	3	3	50	9
Low CTC	4	36	10	p value=0.037*
Presence of CTM	2	3	40	4.8
Absence of CTM	5	36	12.2	p value=0.1599
CEA > 5 ng/mL	4	20	16.7	1.3
CEA ≤ 5 ng/mL	3	19	13.6	p value=1.0
CA19-9 > 37U/mL	3	7	30	3.4
CA19-9 ≤ 37U/mL	4	32	11.1	p value=0.1632
High Exosome miR-21<br>High Plasma miR-21	5	3	62.5	30
Others	2	36	5.3	p value=0.0008***
High Exosome miR-21<br>High Plasma miR-21<br>High CTC	3	1	75	28.5<br>p value=0.0086**
Others	4	38	9.5	
High Exosome miR-21<br>High Plasma miR-21<br>With CTM	1	0	100	N/A
Others	6	39	13.3	
High Exosome miR-21<br>High Plasma miR-21<br>CEA > 5 ng/mL	4	2	66.7	24.7<br>p value=0.0029**
Others	3	37	7.5	
High Exosome miR-21<br>High Plasma miR-21<br>CA19-9 > 37U/mL	3	2	60	13.9<br>p value=0.0199*
Others	4	37	9.8	

Supplementary methods:

Characterisation and quantification of Exosomes:

Scanning electron microscope (SEM): Isolated exosomes dissolved in PBS were mixed with 4% paraformaldehyde solution and dropped on a holder. Fixed samples were then treated with a series of solutions with different concentrations of alcohol (25%, 50%, 75%, 100%) to be preliminarily dehydrated. After that, samples were freeze-dried for further dehydration before final examination by High resolution thermal field emission scanning electron microscope (HRFEG-SEM, JSM-7610F, JEOL, Japan) in National Tsing Hua University.

Nanoparticle tracking analysis (NTA): To study the size of the isolated exosomes, nanoparticle tracking analysis on the NanoSight NS300 (Malvern, UK) was used and analyzed with ZetaView.

Western blot: Briefly, resuspended exosomes were mixed with sample buffer to extract proteins which were further separated by SDS-PAGE and transferred to nitrocellulose membranes. Target proteins were probed by specific primary antibodies and visualised by fluorescent secondary antibodies after incubations. Antibodies used in detection are Alix (E6P9B; CST), HSP70(D69; CST), PD-L1(E1L3N; CST), Calreticulin (D3E6; CST), CD63(CBL553; MK), CD81(TAPA-1; BLG), CD9(D8O1A; CST), GAPDH (14C10; CST), Goat α Rabbit IgG(H+L) (115-035-003, JIR). (Experiments of NTA and WB, were both supported by our co-authors at the Institute of Microbiology and Immunology, National Yang-Ming Chiao-Tung University, Taipei, Taiwan)
